# Cancer Patients and Anxiety: A Gender Perspective

**DOI:** 10.3390/ijerph17041302

**Published:** 2020-02-18

**Authors:** Paula Parás-Bravo, María Paz-Zulueta, Ester Boixadera-Planas, Víctor Fradejas-Sastre, Domingo Palacios-Ceña, César Fernández-de-las-Peñas, Cristina Alonso-Blanco

**Affiliations:** 1 Faculty of Nursing, University of Cantabria. Avda Valdecilla s/n, 39008 Santander, Cantabria, Spain; paula.paras@unican.es (P.P.-B.); victor.fradejas@unican.es (V.F.-S.); 2 Health Research Institute IDIVAL, Research Nursing Group, Cardenal Herrera Oria s/n, 39008 Santander, Cantabria, Spain; 3 Health Research Institute IDIVAL, Health Law and Bioethics Group. GRIDES, Cardenal Herrera Oria s/n, 39008 Santander, Cantabria, Spain; 4 Ester Boixadera-Planas. 4. Servei d’Estadística Aplicada of the Universitat Autònoma de Barcelona.Plaça Cívica, 08193 Bellaterra, Barcelona, Catalonia, Spain; Ester.Boixadera@uab.cat; 5 Department of Physiotherapy, Occupational Therapy, Rehabilitation, and Physical Medicine, University Rey Juan Carlos, Avda de Atenas s/n, 28922 Alcorcón, Madrid, Spain; domingo.palacios@urjc.es (D.P.-C.); cesar.fernandez@urjc.es (C.F.-d.-l.-P.); cristina.alonso@urjc.es (C.A.-B.); 6 Research Group of Humanities and Qualitative Research in Health Science of University Rey Juan Carlos, Avda de Atenas s/n, 28922 Alcorcón, Madrid, Spain

**Keywords:** cancer, gender, anxiety, pain, side effects

## Abstract

The complexity of the diagnosis and treatment of cancer means that it is often associated with anxiety symptoms. The aim of our study was to further our understanding of the oncological process and the presence of anxiety symptoms, from a gender perspective. A cross-sectional study was performed, examining 402 medical records obtained by simple random sampling of oncology patients at a hospital in northern Spain from July 2012 to July 2014. Data collection took place between February and May 2015. Psychiatric and sociodemographic variables were gathered, as well as pain variables and information regarding the oncological process. The data analysis included a descriptive univariate analysis and a bivariate analysis, and a logistic regression model was performed. Our results suggest that women with cancer suffer more anxiety symptoms than men with cancer. Women with anxiety symptoms represented 76.5% of all patients with anxiety. The OR of suffering anxiety symptoms between women and men was 2.43 (95% CI 1.05–5.63) (*p* = 0.04). A greater incidence of anxiety symptoms was found in patients with cancer pain and oncological treatment with biological therapy. Our results suggest that the gender perspective is necessary in the management of mental health in patients with cancer. Nonetheless, further studies are necessary to confirm our findings.

## 1. Introduction

In 1946, the World Health Organization (WHO) defined overall health as “a complete state of physical mental and social well-being and not only the absence of illness or disease” [1]. With regards to this definition, we can conclude that mental health is an inherent part of physical health and that it is practically impossible to feel healthy if we are lacking psychological wellbeing, although this is also intimately related with our circumstances and our ability to adapt to these.

Although a large proportion of the global population suffers from mental disorders, it is difficult to calculate their impact at an epidemiological level, as mental disorders are frequently underdiagnosed. The Mental Health Action Plan 2013–2020 published by the WHO [2] gathers the most relevant epidemiological data regarding mental disorders. The WHO estimates that one out of every four people, i.e., 25% of the worldwide population, will suffer a mental disorder during their lifetime. Currently, 450 million people suffer from a mental illness.

The epidemiological magnitude of cancer also represents one of the largest public health burdens. The Globocan 2018 database reported 18.1 million new cases in the year 2012, estimating that, in 2040, this will increase to 29.5 million new cancer diagnoses worldwide [3]. The complexity of the oncological process, the diagnosis, treatment, secondary effects, and pain, among other aspects, are all associated with greater emotional distress [4]. 

The magnitude of these pathological issues leads us to question how cancer and mental disorders interact when they coexist, and indeed, many scientific publications have studied this relationship [5,6,7]. Thirty percent of oncological patients are estimated to suffer emotional distress during treatment [5,6,7,8]. Linden et al, reported that, on average, 19% of people with cancer present anxiety symptoms, and this figure is twice as frequent in women. 

Considering the most prevalent types of cancer, a level of clinical anxiety was reported in 7.5% of men with prostate cancer, increasing to almost 28.4% of women with gynecological cancer [5]. However, according to Watts et al, levels of anxiety in men with prostate cancer can reach up to 27.04% prior to receiving treatment, decreasing to 15% during treatment and to 18.5% after completing treatment [6]. In contrast, Maas et al, studied anxiety in women with breast cancer, reporting a prevalence ranging between 17.9% and 33.3% [7].

In this context, our aim was to further our understanding of anxiety symptoms and the oncological process from a gender perspective, among a sample of oncological patients from a hospital in northern Spain.

## 2. Materials and Methods 

The study protocol was approved by the Cantabria Clinical Research Ethics Committee on 29 May 2014; this was prior to data acquisition, which began in February 2015. The relevant authorization was also obtained in line with hospital regulations. The data were treated anonymously and confidentially under Spanish Organic Law 15/1999 of 13 December on Personal Data Protection [9]. Because the data were analyzed anonymously, obtaining patient consent to research their data was deemed unnecessary. 

### 2.1. Study Design and Participants

**Figure 1:**
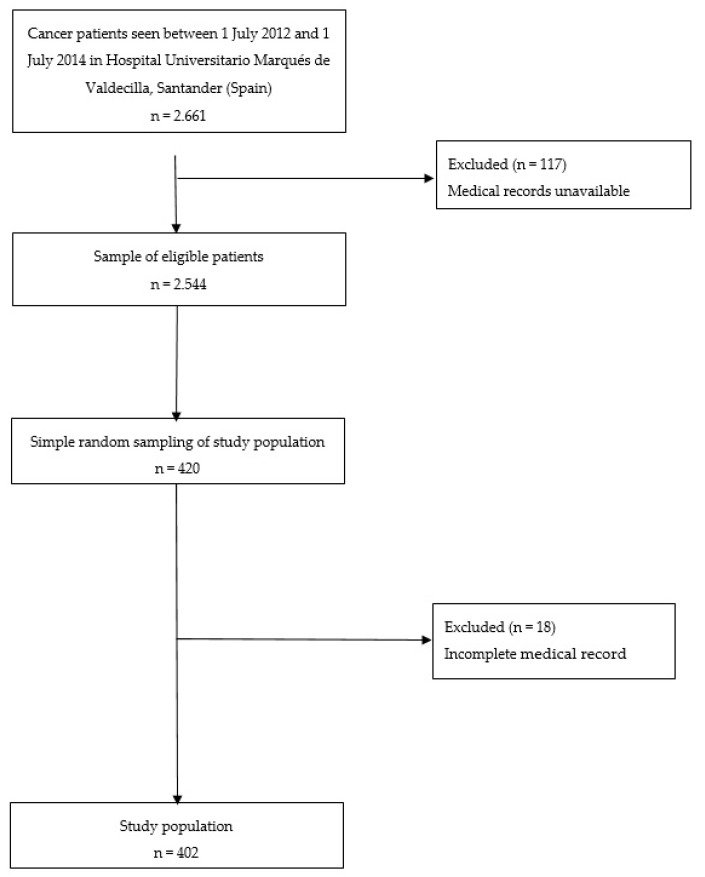
Overall study flow.

A cross-sectional study was performed. The eligibility criteria were patients aged over 18 years with any type of cancer (oncological and/or hematological malignances), of both sexes, and treated at the Oncology Unit of Hospital University Marqués of Valdecilla (National Public Health System of Spain) in northern Spain between 1 July 2012 and 1 July 2014. In total, 420 Oncology Unit patients were selected by simple random sampling from 2661 in total (Figure 1). We obtained the information of the medical records (medical records and nursing assessments). We considered the medical records incomplete when information about all anxiety related variables was missing. We considered the records complete if they contained information about >80% of the remaining variables. If the medical records were incomplete, we withdrew that patient from the study. As a result, of the 420 original subjects, records from 18 patients (4.3%) were removed.

The following criteria were used to determine the sample size. Regarding prevalence rates, the literature available is scarce, therefore, the sample size was calculated by assuming maximum indeterminacy (*p* = 0.5). Under this assumption, with a 95% confidence interval for a maximum error of 5%, a sample of 400 patients was estimated. 

### 2.2. Data Sources and Variables

We collected data between February and May 2015. Based on patient medical records and nursing assessments, we obtained information for each cancer patient who underwent active treatment from 1 July 2012 to 1 July 2014. Full oncological and psychiatric details were accessed. The following variables were gathered:

- Anxiety symptoms: patients diagnosed with anxiety symptoms by a clinician specialized in psychiatry, following the criteria of the American Psychiatric Association as stated in the DSM-V [10].
- Sociodemographic variables: age, sex, marital status, number of children, and education level.
- Psychiatric symptoms: psychiatric diagnosis, psychiatric treatment, anxiety crises, and use of relaxation techniques.
- Pain characteristics: presence of pain, location of pain, intensity of pain, and pain control. The presence of pain was treated dichotomously (yes/no). In patients whose pain was treated with analgesics, the type of analgesic, route of administration, and intensity of pain were recorded. The latter was measured following the recommendations of the European Society for Medical Oncology using the Visual Analogue Scale [11], from 0 (‘no pain’) to 10 (‘extreme pain’). Self-perceived pain control, reported by the patients themselves, was recorded in the nursing history; this information was obtained directly from the medical record. The ‘pain control’ variable was categorized into ordinal levels: ‘no pain’, ‘adequate pain control’, and ‘inadequate pain control’. The Oncology Unit followed the most recent recommendations of the European Society for Medical Oncology to determine the prescription of analgesic treatment [11].
- Oncological process characteristics: type of cancer (location), cancer treatment (chemotherapy, radiotherapy, hormone therapy, biological therapy, and/or surgery), and any side effects of cancer treatment. The symptoms considered as being side effects were those established by the National Cancer Institute [12].

### 2.3. Data Analysis

The data analysis included a descriptive univariate analysis and a bivariate analysis of the characteristics by the indicator of Anxiety Symptoms. The bivariate analysis was done globally and also stratified by gender. Mean, Standard Deviation (SD), and Mean Confidence Interval were presented for continuous variables, and the contrast between Anxiety Symptoms of the patients was done with the Wilcoxon two-sample test (W). In case of categorical variables, we presented counts and percentage of the sample, using the Pearson’s Chi-Square test, Likelihood ratio (LR) chi-square test or Fisher’s exact test to contrast differences between groups. 

A logistic regression model was performed to analyze the explanatory variables that influence the variable: Anxiety Symptoms. The selection of variables was carried out by “backward” automatic procedure. We established a logistic regression to assess differences in the probability of suffering Anxiety Symptoms according to the different explanatory variables. The estimated Odds Ratios (OR) between each pair of categories were presented (p values were corrected taking into account the Tukey adjustment for multiple comparison).

All statistical analyses were performed using SAS v9.4 (SAS Institute Inc., Cary, NC, USA) Statistical decisions have been made taking the value 0.05 as a level of significance

## 3. Results

### 3.1. Sociodemographic Characteristics of the Total Sample and According to Anxiety Symptoms and Gender

In total, 402 medical histories were reviewed, of which, 57.5% were women with a mean age of 61.2 [SD 13.1] (See Table 1). The mean age of patients without anxiety symptoms was 61.6 years [SD 12.9] compared to 59.6 years [SD 13.8] in the case of patients with anxiety. Women represented 76.5% (n = 62) of patients with anxiety this finding was statistically significant (*p* < 0.01).

In the case of female patients, in both groups (with and without anxiety symptoms), over 70% of women were married (n = 119; n = 42), between 10% to 15% (n = 22; n = 6) of women were single, and between 5% to 10% were widows (n = 7; n = 6). In both groups, 75% had children. The median was two children. Approximately 65% of female patients (n = 105; n = 38) had a basic education, between 15% to 20% (n = 26; n = 20) had vocational training, and 15% (n = 23; n = 8) had university degrees.

In the case of men, in both groups there were over 70% of married men (n = 103; n = 12), and between 15% to 18% (n = 19; n = 3) of single men. Between 65% to 70% (n = 92; n = 11) of men had children, with a median of two children. Almost half of the male patients (n = 52; n = 7) had basic studies, between 20% to 30% (n = 39; n = 3) had vocational training and almost 30% (n = 34; n = 4) had university studies. 

### 3.2. Psychiatric, Cancer Pain, and Oncological Treatment Characteristics of Patients According to Anxiety Symptoms and Gender 

#### 3.2.1. Psychiatric Characteristics of Patients according to Anxiety Symptoms and Gender

Up to 6.5% (n = 26) of patients had a psychiatric diagnosis, of which, 73.1% (n = 19) also presented anxiety symptoms (See Table 2).

Up to 27.4% (n = 17) of women with anxiety symptoms had a mainly anxious and/or depressive psychiatric diagnosis, and this diagnosis came after the beginning of the peri-oncological process (n = 13; 76.5%). Nonetheless, the percentage of patients who suffered anxiety crises was low (n = 3; 4.8%). Only 12.90% (n = 8) had psychological treatment with a weekly or monthly frequency. Up to 8.06% (n = 5) performed some type of relaxation technique. 

In the case of men, only 10.5% (n = 2) had a psychiatric diagnosis, in all cases this was a depressive type, and the diagnosis was made prior to the peri oncological process. One suffered anxiety crises (n = 1; 5.3%). Only one patient received psychological treatment (n = 1; 5.3%) on a monthly basis. Only one patient performed some type of relaxation technique.

Women presented a greater prevalence of psychiatric diagnoses compared to men (*p* < 0.01).

#### 3.2.2. Pain Characteristics of Patients according to Anxiety Symptoms and Gender

Anxiety symptoms and pain were present in 29% (n = 18) of women and 63.2% (n = 12) of men. This difference was statistically significant compared to patients without pain (*p* < 0.01; *p* = < 0.01) (See Table 2).

The intensity of pain was similar in men and women with and without anxiety symptoms. La Table S1 shows the consumption of analgesics included NSAIDs, other non-opioid analgesics and both weak and strong opioids. Oral administration of analgesics was most frequent. Regarding the need for rescue analgesia, this was required by 61.1% (n = 11) of women and 63.6% (n = 7) of men with anxiety symptoms. 

The effectiveness of the analgesic varied between “total control (45.8%; n = 33) and “partial control” (54.2%; n = 39) of symptoms, almost in equal proportion in both groups (with and without anxiety symptoms). No patients referred a total lack of control of symptoms. 

No statistically significant differences were found in the remaining variables except for the need for rescue analgesia in the case of women (*p* = 0.01).

The location of pain varied, for which 11 different locations were described (See Table S2).

#### 3.2.3. Characteristics of the Oncological Treatment according to Anxiety Symptoms and Gender 

In both groups, women were primarily treated with chemotherapy (91.7% n = 155; 95.2% n = 59) and intravenous administration of it was most frequent (91.6% n = 142; 98.3% n = 58) (See Table 2).

No statistically significant variables were detected in any variable related to the oncological process, except for treatment with radiation therapy in women, where the prevalence of anxious symptoms was significantly greater (*p* < 0.01).

Men were primarily treated with chemotherapy in both groups (90.1% n = 137; 73.7% n = 14) for which intravenous administration of therapy was most frequent (93.4% n = 128; 85.7% n = 12).

Statistically significant differences were found (*p* = 0.04) regarding treatment with chemotherapy in men, where the number of patients without anxiety symptoms and chemotherapy treatment was greater.

The distribution of the oncological diagnoses according to the anxiety symptoms and gender are described in Table S3.

In total, 34 different diagnoses were detected. In women, the most frequent diagnosis was breast cancer with 69 (40.8%) patients without anxiety symptoms compared to 42 (67.7%) patients with anxiety symptoms. In men, the most common diagnosis was rectal cancer with 37 (24.3%) patients without anxiety symptoms and 3 (15.8%) patients with anxiety symptoms.

The secondary effects (Table S4) of the oncological treatments are multiple and diverse. Thirty-five different symptoms were detected. In women, statistically significant differences were observed regarding anxiety symptoms in patients who referred: alopecia (*p* < 0.01), asthenia (*p* < 0.01), arthralgias (*p* < 0.01), mucositis (*p* = 0.02), weight loss (*p* = 0.02), nail toxicity (*p* = 0.04) and insomnia (*p* < 0.01).

### 3.3. Logistic Regression Model

The explanatory variables that were included in the logistic regression were: Gender, Cancer Pain, Biological Therapy, and Location of Cancer. Table S5 shows the Type III Effects. 

Table S6 shows the distribution of anxiety symptoms according with the explanatory variables.

Table 3 shows the odds ratio of the explanatory variables.

Up to 26.8% of women and 11.1% of men suffered anxiety symptoms. The OR of suffering anxiety symptoms between women and men was 2.43 (95% CI 1.05–5.63). This difference was statistically significant (*p* = 0.04).

Up to 15.6% without cancer pain and 40.5% with cancer pain suffered anxiety symptoms. The OR of suffering anxiety symptoms according cancer pain was 6.06 (95% CI 3.13–11.73). This difference was statistically significant (*p* < 0.0001).

Up to 17.3% without biological therapy and 26.6% with biological therapy suffered anxiety symptoms. The OR of suffering anxiety symptoms according oncological treatment with biological therapy was 1.89 (95% CI 1.07–3.32). This difference was statistically significant (*p* = 0.03).

Although there was a statistically significant influence in considering the variable Location of cancer (F = 3.32; *p* = 0.01, see Table S5), there were no statistically significant differences across each pair of locations of cancer. 

## 4. Discussion

Our results suggest that women with cancer suffer more anxiety symptoms than men with cancer. These findings coincide with other authors, such as Linden et al. [5], who also identified greater anxiety in women with cancer, compared to men.

The greater incidence of anxiety in women is not exclusive of oncology. As noted by Donner and Lowry [13], the US National Institute of Mental Health estimates that women have 60% more probabilities of developing an anxiety disorder throughout their life. Indeed, in our study, 27.4% of women with anxiety symptoms presented a prior psychiatric diagnosis and, in most cases, this was of a depressive/anxious type. In contrast, only 10.5% of men presented a previous psychiatric diagnosis. 

It is estimated that 30% of people with cancer suffer some type of emotional distress [5,6,7,8]; if we add the coexistence of pain, the probabilities of suffering anxiety symptoms increase. Almost 20% of our patients suffered pain, which increased up to 30% and 60% in women and men with anxiety symptoms, respectively. Stanton et al. [14], revealed that pain is present in 20% to 50% of patients with cancer. Furthermore, according to Mehnert et al. [15], it can reach up to 90% in the final stages of the disease. In our patients, the presence of pain was significantly greater, both in men and women, in the group with anxiety symptoms. Besides, the OR of suffering anxiety symptoms according cancer pain was higher. This data is logical if we consider the loss of quality of life when suffering pain that is more or less intense over a prolonged period of time. Several publications relate pain with anxiety as this involves an important loss of quality of life of patients with cancer [16,17,18]. On occasion, pharmacological treatments for pain and anxiety can be the same. However, despite the fact that the WHO, the American Cancer Society, and the European Society for Medical Oncology [11,19,20] include the use of psychotropic drugs in their guidelines on the management of oncological pain, consumption of these is not always related with greater pain control in patients with cancer [4]. 

The control of anxiety in cancer patients is particularly difficult considering the complexity of the cancer process, the level of uncertainty, and the fear of death, all of which can generate feelings of anguish in patients. Some of our patients practiced relaxation techniques to control anxiety symptoms. Indeed, following this perspective, Parás-Bravo et al, reported quality of life improvements via an intervention for the reduction of anxiety in cancer patients [16]. 

Lastly, the presence of side effects, mainly due to radiation therapy and chemotherapy, were closely related with anxiety symptoms in women. Some secondary effects also negatively influence body image and the performance of activities of daily living and leisure which can, in turn, increase anxiety symptoms and affect quality of life [21,22]. The OR of suffering anxiety symptoms according oncological treatment with biological therapy was higher. This could be due to the side effects of these therapy.

Powell et al. [23] emphasize the importance of the psychological adaptation to the secondary effects of therapies against cancer, and Kayl and Meyers [24] underline the need to design intervention guidelines to control the adverse effects and reduce the negative impact that these have on the quality of life of patients.

### Limitations

In retrospective studies based on secondary information (registers), the quality of information may be poor due to incomplete records or a lack of agreement between the different records available. For this reason, we purposely selected the homogeneous variables that could be gathered from secondary records. The study protocol included the individual verification of discrepancies (contradictory information) with the doctors and nurses responsible for the patient; we conducted a sensitivity analysis by incorporating the information separately. A 100% agreement was found for the variables under study. 

One of the possible limitations of this study is the lack of statistical power. This means that certain associations may not reach statistical significance. Thus, in the groups with the diagnosis of anxiety symptoms, the sample size of the groups is too small. The analysis included patients with a previous psychiatric diagnosis; however, due to their small number, it was considered statistically irrelevant. 

A further limitation is that the location of the cancer was not homogeneous in our sample. Finally, it should be noted that anxiety may appear as a consequence of cancer treatment.

## 5. Conclusions

The results of our study suggest that gender differences exist regarding the suffering of anxiety symptoms in oncological patients. The women in our sample presented greater anxiety than men. Many factors can influence these symptoms. In the present sample, the most important factors were the presence of pain and oncological treatment with biological therapy. In many fields, the gender perspective is present; however, in health issues, gender is highly relevant due to its repercussions. The WHO recognizes gender as being a determinant of health and with the Strategy on Women’s Health and Well-being in the WHO European Region, the gender perspective is presented as a means to achieve gender equity [25]. Nonetheless, further research is necessary to confirm our findings.

## Figures and Tables

**Table 1:** Sociodemographic characteristics of the total sample and according to the presence of anxiety symptoms.

Characteristics	Total (n = 402)	Anxiety Symptoms		Anxiety Symptoms			
				Female		Male	
	n (%)	No (n = 321)	Yes (n = 81)	No (n = 169)	Yes (n = 62)	No (n = 152)	Yes (n = 19)
**AGE (years), Mean [SD]**	61.2; [SD 13.1] (95% CI 59.9–62.5)	61.6; [SD 12.96] (95% CI 60.2–62.9)	59.6; [SD = 13.8] (95% CI 56.5–62.7)	60.7; [SD 12.4] (95% CI 57.4–63.9)	58.5; [SD 13.6] (95% CI 54.9–62.1)	62.6; [SD 13.5] (95% CI 59.2–65.9)	63.1; [SD 14.4] (95% CI 59.6–66.6)
		*p* = 0.29 ^a^		*p* = 0.32^a^		*p* = 0.67 ^a^	
**Gender**							
Male	171 (42.5%)	152 (47.4%)	19 (23.5%)				
Female	231 (57.5%)	169 (52.7%)	62 (76.5%)				
		*p* < 0.01 ^b^					
**Marital Status**							
Married	276 (68.6%)			119 (78.8%)	42 (72.4%)	103 (79.8%)	12 (70.6%)
Single	50 (12.4%)			22 (14.6%)	6 (10.3%)	19 (14.7%)	3 (17.7%)
Divorced	9 (2.2%)			3 (1.9%)	3 (5.2%)	2 (1.6%)	1 (5.9%)
Widowed	18 (4.5%)			7 (4.6%)	6 (10.3%)	4 (3.1%)	1 (5.9%)
Separated	2 (0.5%)				1 (1.7%)	1 (0.8%)	
				*p* = 0.15^c^		*p* = 0.78^c^	
**Children**	267 (66.4%)			117 (77.5%)	47 (81%)	92 (71.3%)	11 (64.7%)
				*p* = 0.57^c^		*p* = 0.58^c^	
**Number of Children Mean [SD]**	1.8; [SD1.0] (IC95%1.7–1.9)			1.8; [SD 1.0] (95% CI 1.6–1.9)	2; [SD 1.3] (95% CI 1.6–2.4)	1.8; [SD 0.9] (95% CI 1.6–1.9)	2.1; [SD 1.1] (95% CI 1.3–2.9)
				*p* = 0.43 ^a^		*p* = 0.39 ^a^	
**Educational Level**							
Elementary	202 (50.2%)			105 (68.2%)	38 (65.5%)	52 (41.6%)	7 (50%)
Secondary	80 (19.9%)			26 (16.9%)	12 (20.7%)	39 (31.2%)	3 (21.4%)
University	69 (17.2%)			23 (14.9%)	8 (13.8%)	34 (27.2%)	4 (28.6%)
				*p* = 0.81 ^b^		*p* = 0.72 ^c^	

^a^ Wilcoxon Two-Sample Test. ^b^ Chi-Squared Test. ^c^ LR-Chi-Squared Test. SD: Standard Deviation. CI: Confidence Interval.

**Table 2:** Psychiatric, cancer pain, and oncological treatment characteristics of patients according to anxiety symptoms and gender.

Psychiatric, cancer pain and oncological treatment characteristics	Total (n = 402) n (%)	Anxiety Symptoms			
		Female		Male	
		No (n = 169)	Yes (n = 62)	No (n = 152)	Yes (n = 19)
**Psychiatric diagnosis**	26 (6.5%)	5 (2.9%)	17 (27.4%)	2 (1.3%)	2 (10.5%)
		*p* ≤ 0.01 ^a^		*p* = 0.06 ^b^	
**Type of psychiatric diagnosis**					
Paranoid schizophrenia	3 (11.5%)	1 (20%)	1 (6.3%)	1 (50%)	--
Adjustment disorder with anxiety	2 (7.7%)	--	2 (12.5%)	--	--
Adjustment disorder with mixed anxiety and depressed mood	5 (19.2%)	1 (20%)	3 (18.8%)	1 (50%)	--
Adjustment disorder with depressed mood	14 (53.8%)	2 (40%)	10 (62.5%)	--	2 (100%)
Unspecified psychotic disorder	1 (3.8%)	1 (20%)	--	--	--
**Previous psychiatric Diagnosis^c^**	7 (26.9%)	2 (40%)	4 (23.5%)	1 (50%)	--
		*p* = 0.59 ^b^			
**Anxiety crisis**	4 (0.9%)	--	3 (4.8%)	--	1 (5.3%)
**Type of treatment-psychiatric/psychological**					
Psychiatric	--	--	--	--	--
Psychological	9 (100%)	--	8 (100%)	--	1 (100%)
**Frequency Psychiatric/psychological treatment**					
Weekly	3 (33.3%)	--	3 (37.5%)	--	--
Monthly	6 (66.7%)	--	5 (62.5%)	--	1 (100%)
**Relaxation techniques**	6 (1.5%)	--	5 (8.1%)	--	1 (5.3%)
**cancer pain**	74 (18.4%)	16 (9.5%)	18 (29%)	28 (18.4%)	12 (63.2%)
		*p* < 0.01 ^a^		*p* < 0.01 ^a^	
**Intensity** **Mean [SD]^d^**	5.6; [SD1.4] (95% CI 5.3-6)	5.44; [SD1.3] (95% CI 5.2-5.6)	5.61; [SD1.3] (95% CI 5.3-6)	5.61; [SD1.6] (95% CI 5.4-6)	5.92; [SD1.3] (95% CI 5.29-6.6)
		*p* = 0.71 ^e^		*p* = 0.58 ^e^	
**Analgesic USE**	72 (97.3%)	16 (100%)	18 (100%)	27 (96.4%)	11 (91.7%)
		*p* = 0.52 ^f^		*p* = 0.52 ^b^	
**Oncological treatment**					
**chemotherapy**	365 (90.8%)	155 (91.7%)	59 (95.2%)	137 (90.1%)	14 (73.7%)
		*p* = 0.37 ^a^		*p* = 0.04 ^a^	
**Route of administration**					
Endovenous	340 (93.2%)	142 (91.6%)	58 (98.3%)	128 (93.4%)	12 (85.7%)
		*p* = 0.08 ^a^		*p* = 0.29 ^a^	
Oral	75 (20.5%)	27 (17.4%)	10 (16.9%)	35 (25.6%)	3 (21%)
		*p* = 0.94 ^a^		*p* = 0.74 ^a^	
Subcutaneous	3 (0.8%)	1 (0.7%)	--	2 (1.5%)	--
**Radiation therapy**	178 (48.8%)	73 (43.2%)	39 (62.9%)	57 (37.5%)	9 (47.4%)
		*p* < 0.01 ^a^		*p* = 0.4 ^a^	
**Hormonal therapy**	56 (13.9%)	38 (22.5%)	16 (25.8%)	2 (1.3%)	--
		*p* = 0.6 ^a^			
**Biological therapy**	124 (30.8%)	54 (31.9%)	23 (37.1%)	37 (24.3%)	10 (52.6%)
		*p* = 0.46 ^a^		*p* = 0.01 ^a^	
**Surgery**	190 (74.3%)	91 (53.9%)	35 (56.5%)	57 (37.5%)	7 (36.8%)
		*p* = 0.72 ^a^		*p* = 0.95 ^a^	

^a^ Chi-Squared Test. ^b^ Fisher’s Exact Test. ^c^ Psychiatric diagnosis prior to the oncological peri-diagnosis. ^d^ Visual Analogue Scale. ^e^ Wilcoxon Two-Sample Test. ^f^ LR-Chi-Squared Test. SD: Standard Deviation.

**Table 3:** Odds ratio.

Effect	Category 1	Category 2	Contrast					
			t Value	DF	Ajusted *p* Value	OR	95% CI	
**Gender**	Female	Male	2.08	394	0.04	2.43	1.05	5.63
**Cancer pain**	Yes	No	5.37	394	<0.0001	6.06	3.13	11.73
**Biological therapy**	Yes	No	2.21	394	0.03	1.89	1.07	3.32
**Location of cancer**	Ginecological	Gastrointestinal	2.62	394	0.07	3.18	1.34	7.56
		Respiratory	1.97	394	0.28	3.59	1.00	12.84
		Hematological	2.60	394	0.07	3.63	1.37	9.60

## Supplementary Materials

The following are available online at https://www.mdpi.com/1660-4601/17/4/1302/s1, Table S1: Characteristics of treatment of pain of patients according to anxiety symptoms and gender. Table S2: Location of cancer pain. Table S3: Distribution of the oncological diagnosis according to the anxiety symptoms and gender. Table S4: Secondary effects to oncological treatment according to anxiety symptoms and gender. Table S5: Type III effects. Table S6: Distribution of anxiety symptoms based on explanatory variables.
