# Supplementary material for: Cancer Patients and Anxiety: A Gender Perspective
Source: Int J Environ Res Public Health. 2020 Feb 18;17(4):1302. doi: 10.3390/ijerph17041302 (PMC7175312; doi:10.3390/ijerph17041302)
Supplement: Supplementary file 1 [file ijerph-17-01302-s001.pdf]

iQ TRADUCCIÓN CIENTÍFICA
Isabel Victoria Quintero Hodgson
Calle Pozo Nuevo 23, Bajo A
28430, Alpedrete, Madrid
Tel.: +34 625 134 172
www.iqtraduccion.com
Info@iqtraduccion.com

Date: February 09, 2020

CERTIFICATE OF PROFESSIONAL PROOFREADING

To whom it may concern

This is to certify that the paper entitled *" Cancer Patients and Anxiety: a gender perspective",* submitted for publication as an original research article in IJERPH, has been edited for proper English language, grammar, punctuation, spelling, and overall style by myself and another highly qualified native English editor in my team.

Sincerely,

Isabel Victoria Quintero Hodgson
